# Immunogenic cell death mediation patterns reveal novel paradigm for characterizing the immune microenvironment and immunotherapeutic responses in bladder cancer

**DOI:** 10.3389/fgene.2022.1035484

**Published:** 2022-10-25

**Authors:** Jialei Fu, Wei Zhang, Tao Jiang

**Affiliations:** ^1^ Department of Radiation Oncology, The Affiliated Hospital of Qingdao University, Qingdao, China; ^2^ Department of Urology, Beijing Hospital, National Center of Gerontology, Institute of Geriatric Medicine, Chinese Academy of Medical Sciences, Beijing, China

**Keywords:** bladder cancer, immunogenic cell death, immunotherapy, tumor immune microenvironment, immune checkpoint inhibitors

## Abstract

**Background:** Immunogenic cell death (ICD) plays an important role in several malignancies. However, the role of ICD-mediated patterns in bladder cancer (BCA) remains unknown.

**Methods:** For assessing the ICD-mediated patterns based on the expression of IRGs, 4 large BCA cohorts were obtained. The ICD-mediated patterns of individual samples were quantified as an ICD score by principal component analysis. The correlations of the ICD-mediated patterns with the tumor immune microenvironment (TIME) and responses to immunotherapy were comprehensively evaluated. The IRGs with predictive prognostic values were further validated by *in vitro* loss of function assays.

**Results:** Two distinct ICD-mediated patterns were established, showing distinct clinical features and immune microenvironment features. Although ICD cluster A was associated with a poor prognosis with a high ICD score, it showed an immune activation state with a more favorable response to immunotherapy and treatment that induced ICD. The ICD-related gene, CALR, was significantly upregulated in the T24 BCA cell line relative to the control SV-HUC-1 cells. Knocking down CALR suppressed T24 cell viability and caused ER stress.

**Conclusion:** We identified the existence of distinct ICD-mediated patterns in BCA closely associated with the remodeling of the TIME. Further in-depth examination of ICD-related features is warranted to obtain a broader prospect for therapeutic innovations and improved prognosis of BCA.

## 1 Introduction

Bladder cancer (BCA) is the ninth most frequently diagnosed cancer worldwide with high morbidity and mortality (Antoni et al., 2017). Bladder urothelial carcinoma is categorized as non-muscle invasive BCA (NMIBC) and muscle-invasive BCA (MIBC), with the former accounting for 80% of initial BCA cases, showing better prognoses with surgical resection as the primary treatment (Berdik, 2017). However, the challenge of recurrence remains (Abdollah et al., 2013). The mainstay of treatment for MIBC is platinum-based neoadjuvant chemotherapy combined with radical resection surgery (Witjes et al., 2021). Nevertheless, MIBC patients have a high chance of recurrence and metastasis and therefore a poor prognosis. Immunotherapy, especially immune checkpoint inhibitors (ICIs), is a research hot spot in cancer treatment. Several anti-PD-1/PD-L1 agents have been used in the clinic with definitive efficacy (Powles et al., 2014; Feld et al., 2019). Avelumab and various anti-PD-1/PD-L1 agents have been approved as second-line therapies for advanced/metastatic BCA (Katz et al., 2017). However, notably, the levels of PD-1/PD-L1 expression are closely related to the response rate to therapy, and less than half of MIBC patients achieve a satisfactory prognosis (Gómez de Liaño Lista et al., 2020). Although ICIs are promising for patients with MIBC, the discovery of potential predictors of treatment responses is necessary.

Immunogenic cell death (ICD) is a form of regulated cell death that, wherein a class of signaling molecules called damage-associated molecular patterns (DAMPs) are released when cells undergo death in response to an external stimulus; DAMPs are recognized by antigen-presenting cells and thereby activate an adaptive immune response, ultimately triggering cell death (Galluzzi et al., 2020; Kroemer et al., 2022). Previous studies suggest that chemotherapy, radiotherapy and some targeted anticancer drugs can induce ICD (Rodriguez-Ruiz et al., 2020). Restoring or reinforcing the ICD of tumors for the treatment of cancer has attracted increasing attention and some attempts have been made in this direction. For example, assisting the treatment of BCA by inducing ICD is a promising avenue (Oresta et al., 2021; Nikolos et al., 2022). Additionally, several studies confirm that combination therapy of chemotherapy with ICIs by inducing ICD shows better efficacy (Rodriguez-Ruiz et al., 2020). The reason may be that ICD induced by chemotherapy is mediated by integrated stress response (ISR), which can upregulate the PD-L1 expression (Suresh et al., 2020). Therefore, the synergistic effects of ICD with immunotherapy also deserve further in-depth elucidation. To date, no study has yet revealed the different expression signatures of ICD-related genes (IRGs), if any, in BCA and their association with prognosis and treatment responses.

Our overall analysis of IRGs in BCA establishes two distinct ICD-mediated patterns, which were found to be associated with the tumor immune microenvironment (TIME) and could predict the efficacy of immunotherapy and induction of ICD therapy. The findings highlight the key role of ICD in the TIME of BCA and provide new ideas for further elucidating the mechanism of ICD in BCA and developing innovative intervention strategies.

## 2 Methods

### 2.1 Public datasets and preprocessing

The BCA transcriptome data and corresponding clinical information were obtained from two databases, namely The Cancer Genome Atlas (TCGA) and the Gene Expression Omnibus (GEO). Four different BCA cohorts, including TCGA-BLCA, GSE13507, GSE31684 and GSE32894, were analyzed in the study. Background adjustment for data matrix from affymetrix platforms was performed using the R packages “Affy” and “simpleaffy”. For data matrices from other platforms, normalized sources were downloaded. To ensure the consistency and comparability of TCGA and GEO datasets, transcriptome data of TCGA-BLCA were converted from FPKM to TPM format before data analysis. Additionally, somatic mutation and copy number variation (CNV) data for TCGA-BLCA were downloaded from the TCGA database.

### 2.2 Merging datasets

The “normalizeBetweenArrays” function of the R package “limma” was executed to normalize the expression data for non-uniform matrix distribution. When multiple probes represented the same gene symbol, the mean value was considered as the level of expression. To improve the reliability, the four datasets were merged and batch effects removed using the “combat” function of the R package “sva”.

### 2.3 Unsupervised clustering of IRGs in the merged dataset

A total of 28 IRGs were identified in the merged dataset, including ATG5, BAX, CASP8, ENTPD1, FOXP3, IL10, NT5E, CALR, CASP1, CD4, CD8A, CXCR3, EIF2AK3, HSP90AA1, IFNG, IFNGR1, IL17RA, IL1B, IL1R1, IL6, LY96, MYD88, NLRP3, P2RX7, PIK3CA, PRF1, and TLR4 and TNF. Subsequently, the potential significant prognosis-related genes (*p* < 0.05) were selected by univariate Cox regression analysis, and subjected to unsupervised cluster analysis using the R package “consensusclusterplus”. To ensure the reliability of ICD clustering, the process was iterated 1,000 times. Finally, Kaplan Meier survival curves for different ICD clusters were plotted using the “ggsurvplot” function in the R package “survminer” to in turn validate the predictive value of the prognostic ICD clusters.

### 2.4 Gene set variation analysis, gene ontology annotation and kyoto encyclopedia of genes and genomes analysis

Gene Set Variation Analysis (GSVA) was performed between the ICD clusters using the R package “gsva” to identify the biological differences between the ICD mediation patterns. “c5.go.v7.5.1.symbols” and “c2.cp.kegg.v7.5.1.symbols” served as the reference; the adjusted *p*-value threshold was set at 0.05. Differentially expressed genes (DEGs) between different ICD Mediated Patterns were identified using the R package, “clusterprofiler” (adjusted *p*-value < 0.001 and | logFC | > 1). Gene ontology (GO) annotation and kyoto encyclopedia of genes and genomes (KEGG) pathway analyses were performed using the R package “clusterprofiler” for significant DEGs between different ICD mediation patterns.

### 2.5 Evaluation and classification of the ICD signature

The prognostic value of DEGs between different ICD-mediated patterns was assessed by univariate Cox regression analysis (*p* < 0.05) with the R package “survival”. Unsupervised cluster analysis of genes with significant prognostic values was then performed using the R package “ConsensusClusterPlus”. Next, Kaplan Meier survival analysis for different gene clusters was performed using the “ggsurvplot” function of the R package “survminer”. All DEGs with significant prognostic values were further subjected to PCA to develop an ICD-mediated signature. An ICD score was assigned to each sample using the formula: ICD score = ∑ (principal element 1_e_ + principal element 2_e_), where e is defined as the expression of IRGs. The assignment of the sample to the high- or low-risk ICD group was based on the median ICD score.

### 2.6 Multiomic features of the ICD signature

The R packages “limma” and “ggpubr” were used to compare the differences between ICD scores of ICD and gene clusters. Kaplan-Meier survival analysis for high- and low-risk ICD score groups was performed to evaluate the predictive value of the ICD score for survival. The correlation of the ICD signature with tumor mutation burden (TMB) and oncogenic mutations was analyzed using the R packages “ggplot2” and “ggpubr”. The utility of the ICD signature for different clinical features (gender, age and T stage) was also performed using the R package “ggplot2”.

### 2.7 Predictive value of ICD signature for immunotherapeutic responses

Based on the natural association of ICD with immune function, the relevance of tumor cell ICD signature with immune function and microenvironment was examined and the predictive value of ICD signature for immunotherapeutic efficacy was analyzed. First, the stromal, immune and ESTIMATE scores were calculated for each sample using the R package “ESTIMATE”, and the survival differences between low- and high-ESTIMATE score groups and low- and high-ICD score groups were simultaneously compared. Moreover, the differences in the degree of immune cell infiltration based on the different ICD-mediated patterns were evaluated by single sample gene set enrichment analysis (ssGSEA). The differences in the expressions of immune checkpoint blockade genes (CTLA-4, PD-1 and PD-L1) and immune suppressive cytokines (IL-10, TGF- β2 and TGF- β3) between the high- and low-ICD score groups were evaluated using the R package “limma”. Sensitivity analyses for traditional chemotherapeutic agents and molecular targets were performed using the R package “pRRophetic” according to the gene expression in different ICD clusters to predict pharmacotherapeutic responses. Further, the relationship of the ICD score with the immunotherapy score was predicted based on immunotherapy cohort data from The Cancer Immunome Atlas (TCIA) database (https://www.tcia.at/home) and the correlations of the four immunotherapy strategies (anti-CTLA-4, anti-PD1, anti-CTLA-4 + anti-PD1 and no medication) with different ICD score groups were analyzed.

### 2.8 Cell culture

The SV-HUC-1 (CL-0222) and T24 (CL-0227) cell lines were purchased from Procell Life Science & Technology Co., Ltd. SV-HUC-1 and T24 cells were cultured in Ham’s F-12K medium (Gibco) and RPMI-1640 medium (Gibco), respectively. The media were supplemented with 10% fetal bovine serum (Gibco) and 1% penicillin-streptomycin solution (Gibco). Cells were maintained in an incubator at 37°C with 5% carbon dioxide and 95% relative humidity. Passages were performed when cell confluency reached 80%. Cells in the logarithmic growth phase were used for experiments.

### 2.9 Real-time quantitative reverse transcription PCR

Total cellular RNA was extracted with TRIzol reagent (Invitrogen)and cDNA synthesis was performed with HiScript^®^ II Q RT SuperMix (Vazyme). The relative mRNA levels were quantified *via* real-time quantitative reverse transcription polymerase chain reaction (qRT-PCR) using the 2 × RealStar Fast SYBR qPCR Mix kit (GenStar) on the 7,500 real-time PCR system (Applied Biosystems). β-Actin was used as the reference gene. Primer sequences used in this study were listed in Table 1.

**TABLE 1:** Primers.

Gene(human)	Forward primer	Reverse primer
β-Actin	CTACCTTCAACTCCATCA	GAG​CAA​TGA​TCT​TGA​TCT​TC
CALR	CTC​TGT​CGG​CCA​GTT​TCG​AG	TGT​ATT​CTG​AGT​CTC​CGT​GCA​T
HSPA5	CTC​TGC​CTC​ACC​TCG​CTC​CA	TCG​CAA​TAG​CAA​TGC​CAA​TC
DDIT3	ACCAGGAAACGGAAACAG	TCACCATTCGGTCAATCA
BAX	TTT​TGC​TTC​AGG​GTT​TCA​TC	GAC​ACT​CGC​TCA​GCT​TCT​TG
BCL-2	GCC​TTC​TTT​GAG​TTC​GGT​GG	GAA​ATC​AAA​CAG​AGG​CCG​CA
CD47	TCC​GGT​GGT​ATG​GAT​GAG​AAA	TCC​GGT​GGT​ATG​GAT​GAG​AAA

### 2.10 Cell transfection protocol

The siRNA for CALR was synthesized by Genepharma Biotechnology (Shanghai, China). T24 cells were cultured in a complete medium without antibiotics. Cells were transfected with lipofectamine-2000 following the manufacturer’s instructions, and the gene expression of CALR was determined by qRT-PCR analysis 48 h after transfection.

### 2.11 CCK-8 assay

CCK-8 assay was performed to detect cell viability. T24 cells were incubated in 96-well plates for 72 h after transfection and the culture was continued for another 24 h. Each well was incubated after the addition of CCK-8 solution (10 μl) for 1 h. Finally, the OD at 450 nm was measured using a multifunctional microplate reader (Thermo).

### 2.12 Statistical analysis

DEGs between different groups were compared using the R package “limma”. Categorical variables were compared using the chi-square test. Differences between the groups were analyzed using the student *t*-test. Differences between three or more groups were compared using a one-way analysis of variance (ANOVA). All statistical analyses were performed on R (version 4.1.3, Vienna, Austria) or IBM SPSS (version 22.0, NY, United States). All graphic representations were on GraphPad Prism (version 8.0). Data for all *in vitro* tests were the results of three independent replicates.

## 3 Results

### 3.1 Genetic alterations in IRGs in BCA

**FIGURE 1:**
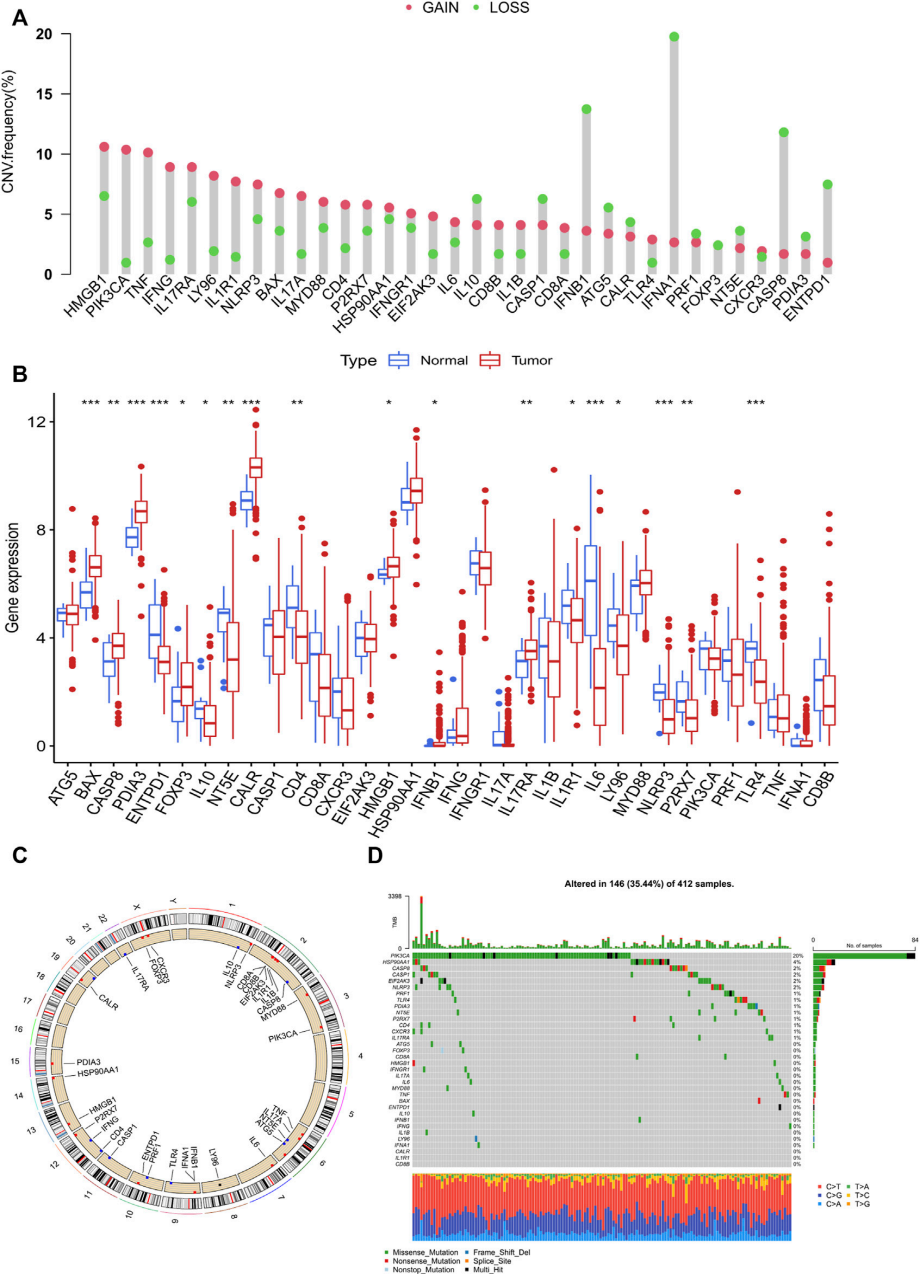
Landscape of genetic variations in IRGs in bladder cancer. **(A)** CNV distributions of IRGs in bladder cancer. **(B)** Different expressions of IRGs in tumour and normal tissues. **(C)** CNV locations of IRGs on 23 human chromosomes. **(D)** Waterfall plot demonstrating the somatic mutation status of IRGs in bladder cancer. Each column represents a single sample and the upper bar graph represents the TMB value. The number on the right represents the frequency of somatic mutations.

A total of 34 IRGs were analyzed (Garg ad et al., 2015) and the gene list is provided in Supplementary Table S1. Preliminary analyses were performed for CNV and somatic mutation data of IRGs in the TCGA-BLCA cohort (Supplementary Table S2, S3). Among the IRGs, the majority (22/34) were characterized by copy number amplification, while a small proportion (12/34) showed copy number deletions (Figure 1A). Moreover, most IRGs showed significant differences in expression between normal and bladder tumor issues (Figure 1B). Additionally, some IRGs with copy number amplification (HMGB1, IL17RA, and BAX) showed increased expression in tumor tissues, while some IRGs with copy number deletions (IL10, NT5E, and ENTPD1) showed decreased expression in tumor tissues. CNV locations on chromosomes of IRGs are annotated in Figure 1C **.** Somatic mutations in the TCGA-BLCA cohort indicated that 35.44% of the samples harbored mutations in IRGs (Figure 1D). The heterogeneity in CNV and somatic mutations in the TCGA-BLCA cohort suggested that BCA may exhibit heterogeneous expression features of IRGs.

### 3.2 ICD-mediated patterns based on 28 IRGs

**FIGURE 2:**
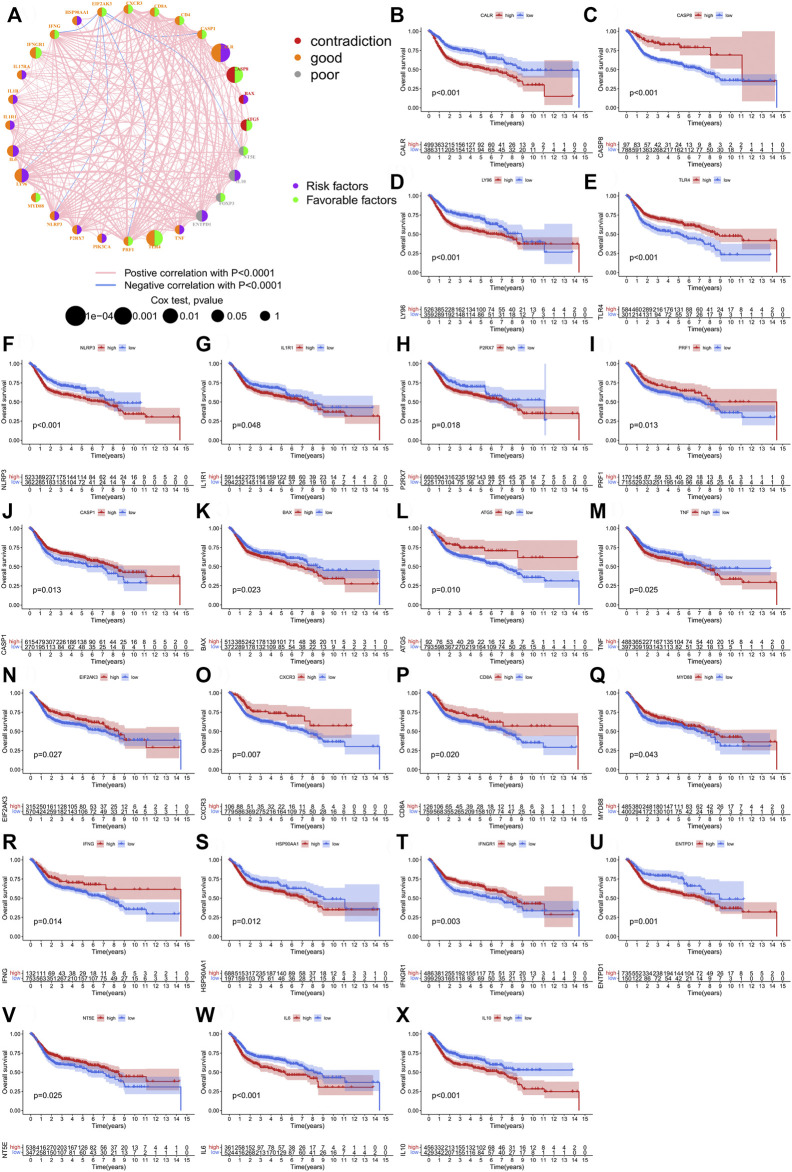
The prognostic role of IRGs in the merged cohort. **(A)** A network illustrating interactions between 28 IRGs. **(B–X)** Kaplan-Meier analysis of 23 IRGs with prognostic roles in bladder cancer in the merged cohort.

Three GEO BCA datasets (GSE13507, GSE31684, and GSE32894) were merged with the TCGA-BLCA cohort (Supplementary Table S4). Expression data for the 28 IRGs were extracted from the merged dataset (Supplementary Table S5). Significant correlations were found among the 28 IRGs (Figure 2A). CALR, TLR4, LY96 and CASP8 could be the key factors at the core of the interactions. Kaplan-Meier survival analysis for the IRGs revealed that the vast majority of genes (23/28) had significant predictive prognostic value (Figures 2B–X). The R package “ConsensusClusterPlus” was utilized to classify the samples of the merged dataset into two different ICD clusters based on the levels of expression of 28 IRGs (Supplementary Figures S1A, Supplementary Table S6). Moreover, the two ICD clusters showed significantly different prognostic predictive outcomes (Supplementary Figure S1B). Supplementary Figure S1C demonstrated the correspondence between ICD clusters and clinical features. Additionally, ICD cluster A could be ideally distinguished from ICD cluster B based on PCA (Supplementary Figure S1D).

### 3.3 GSVA and ssGSEA for ICD clusters

**FIGURE 3:**
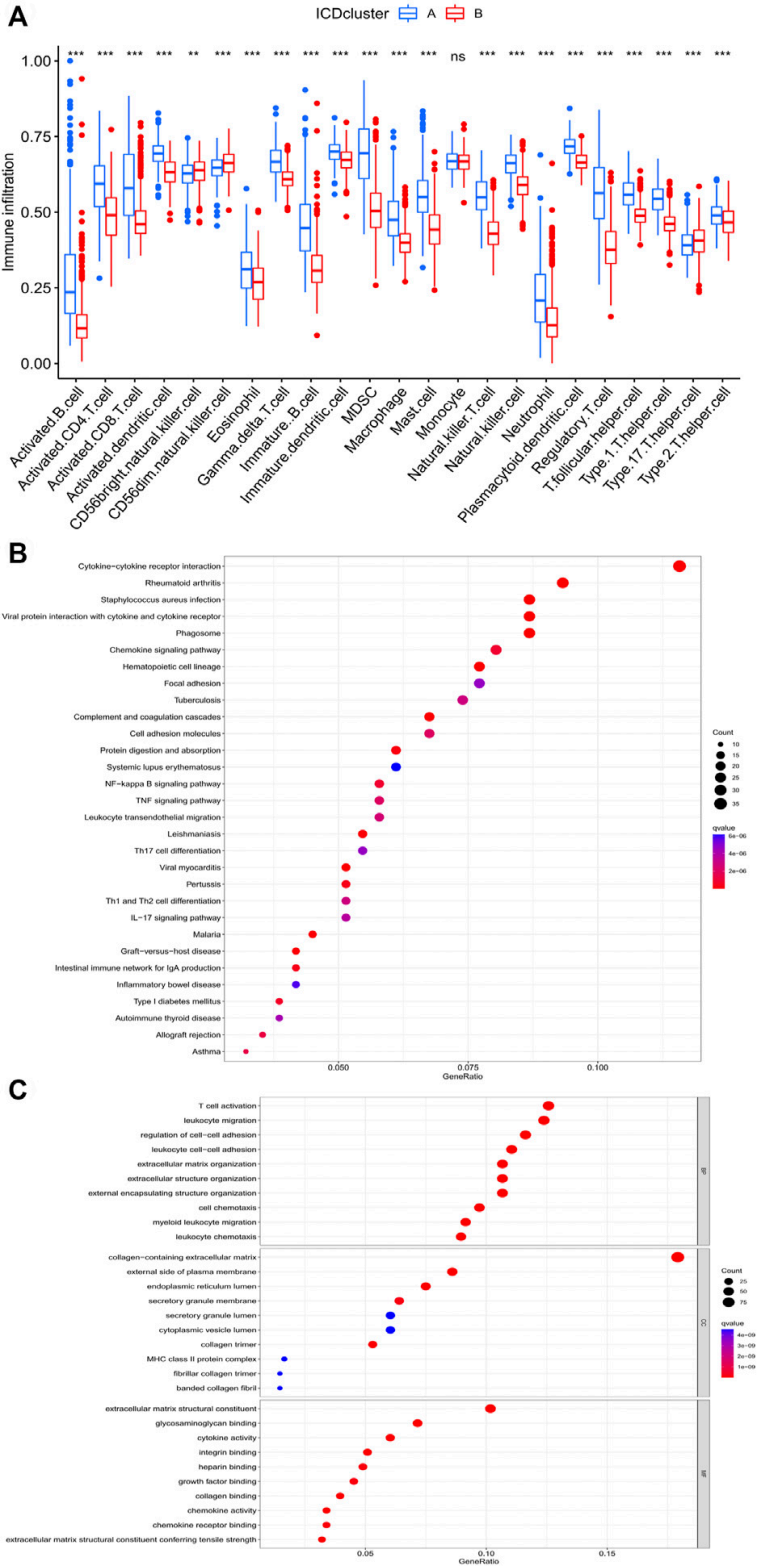
GO, KEGG and ssGSEA analyses based on DEGs in distinct ICD clusters. **(A)** ssGSEA analysis of two distinct ICD clusters. **(B)** Bubble chart presenting KEGG enrichment analysis. **(C)** Bubble chart presenting GO enrichment analysis. The asterisk symbol indicated the statistical *p*-value. (*p < 0.05; **p < 0.01; ***p < 0.001).

To fully probe the biological differences between the ICD clusters, GSVA and ssGSEA were performed. First, the R package “GSVA” was used to identify the KEGG pathways and GO terms related to the ICD characteristics in the merged dataset. These results showed that signaling pathways associated with EMT and migratory abilities of malignant tumors were significantly enriched in ICD cluster A, including ECM receptor interaction, focal adhesion, cytokine receptor interaction and cell adhesion molecules (CAM). Moreover, key signaling pathways involved in inflammation and cancer, NOD-like receptor and JAK-STAT signaling pathways, were also highly enriched in ICD cluster A. Chemokine signaling pathways and leukocyte trans-endothelial migration pathways that regulate the TME by coordinating immune cell trafficking tropism were also enriched in ICD cluster A (Supplementary Figure S2A; Supplementary Table S7). Similarly, almost all GO terms enriched in ICD cluster A corresponded to various immune cell functions, including chemotaxis, activation and migration (Supplementary Figure S2B; Supplementary Table S8). The results of GSVA revealed that the remodeling of tumor inflammatory microenvironment and TIME may be responsible for the poorer prognosis in ICD cluster A. ssGSEA revealed significant differences between the ICD clusters for all 22 immune cell types except monocytes (Figure 3A). Only three immune cell types showed increased infiltration in the ICD cluster B, including CD56^bright^/CD56^dim^ NK cells that dominate the direct tumor-killing effects and T helper 17 T cells, exerting an indirect tumor clearance effect by recruiting the killer cells (Bowers et al., 2017). These three cell types may be involved in enhancing the survival of patients in the ICD cluster B. The vast majority of immune cells showed increased infiltration in ICD cluster A, including not only immune cells (macrophages and mast cells) associated with poorer prognosis in BLCA but also those associated with better prognosis (type 1 T helper cells, regulatory T cells and activated CD8 T cells). A total of 551 DEGs were identified between the ICD clusters (Supplementary Table S9). The results of GO enrichment analysis for DEGs showed that biological processes related to TIME remodeling were significantly enriched, including activation, chemotaxis, migration and adhesion processes of various immune cells, as well as remodeling of the extracellular matrix (Figure 3B). Similarly, KEGG pathway enrichment analysis for DEGs also suggested the overrepresentation of various pathways related to the regulation of immune functions, including cytokine-cytokine receptor interaction, viral protein interaction with cytokine and cytokine receptor, chemokine signaling pathway, phagosome, NF-kappa B signaling pathway, TNF signaling pathway and leukocyte transendothelial migration (Figure 3C).

### 3.4 ICD genetic identification

**FIGURE 4:**
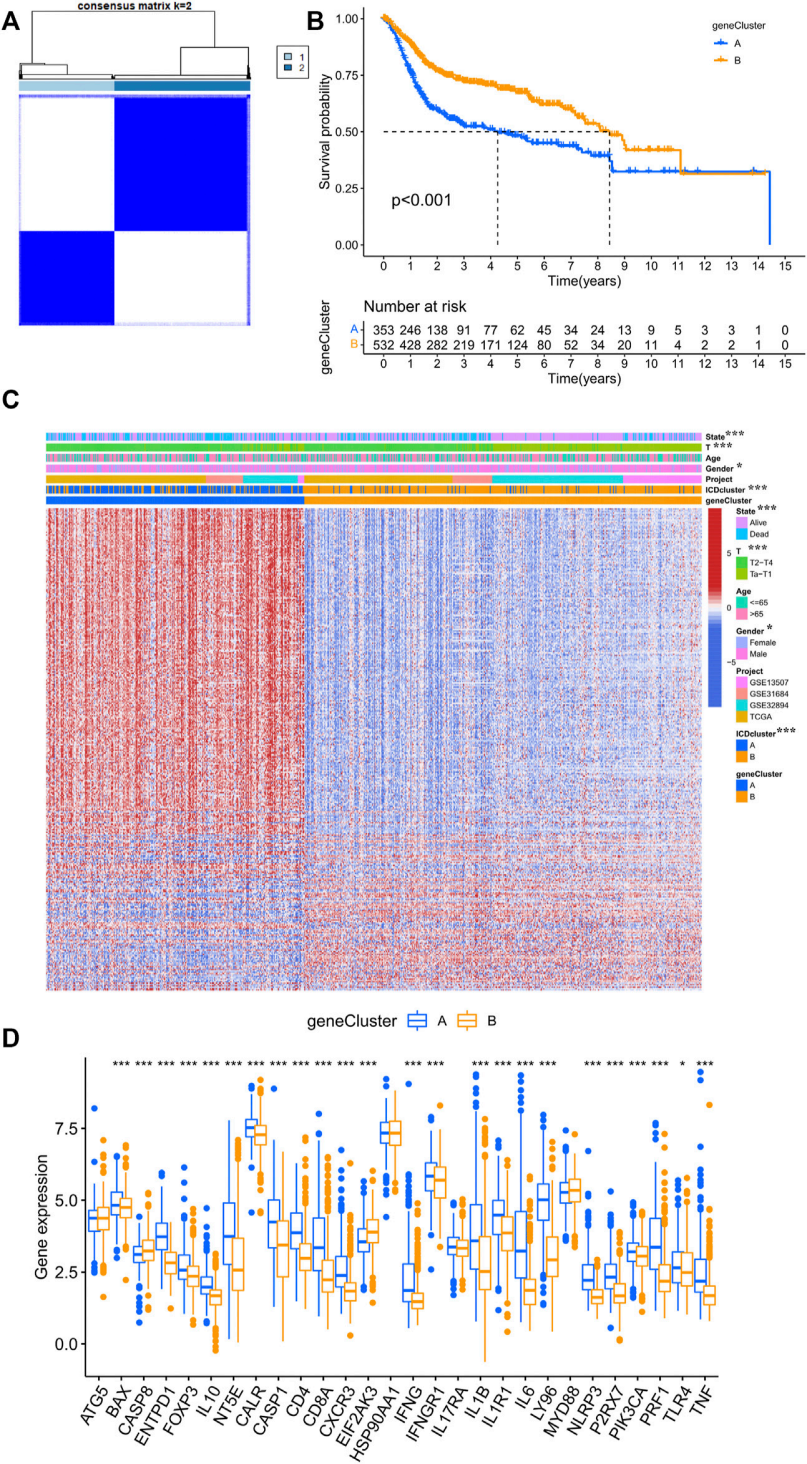
ICD gene clusters in the merged cohort. **(A)** Consensus clustering matrix for k = 2. **(B)** Kaplan–Meier curve survival analysis among two distinct gene clusters. **(C)** Heatmap demonstrating various clinicopathological features of two distinct gene clusters. **(D)** Different expression levels of 28 IRGs in distinct gene clusters. The asterisk symbol indicated the statistical *p*-value. (*p < 0.05; **p < 0.01; ***p < 0.001).

**FIGURE 5:**
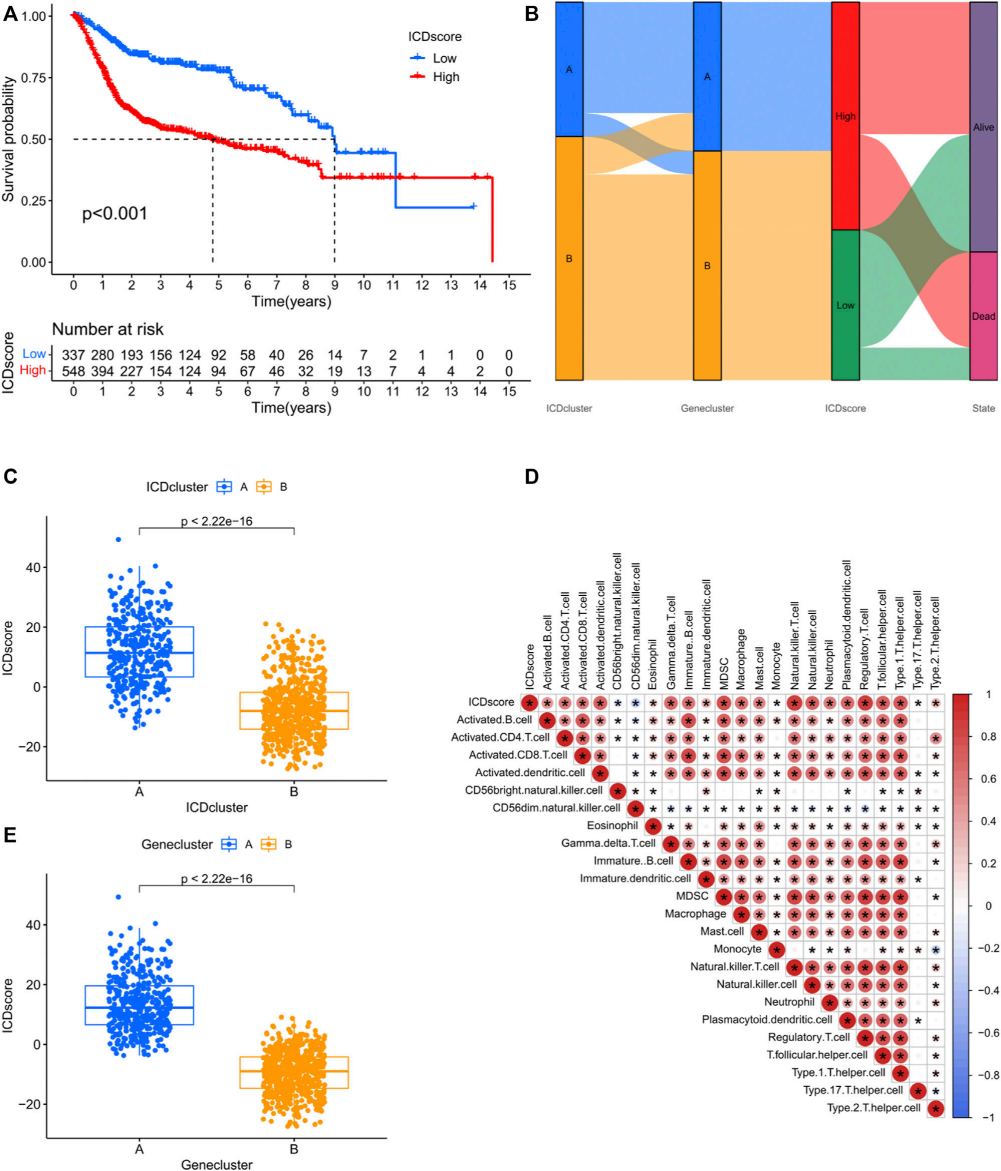
ICD score is a quantification indicator of individual samples in the merged cohort. **(A)** Kaplan–Meier curve analysis of different ICD score groups. **(B)** Sankey diagram demonstrating correlations among ICD clusters, ICD score and ICD gene clusters. **(C)** Differences in ICD scores among two ICD clusters in the merged cohort. **(D)** Differences in ICD scores among two gene clusters in the merged cohort. **(E)** ssGSEA analysis showing a correlation between ICD score and the infiltration abundance of various immune cell.

The results of survival analysis based on DEGs from different ICD clusters indicated that most of them (63.9%) had significant prognostic values (Supplementary Tables S10, S11). To decipher the genetic differences mediated by different ICD clusters, an unsupervised clustering analysis was performed based on the prognostic DEGs. Gene cluster A and B were found in different ICD clusters (Figure 4A; Supplementary Table S12). The ICD cluster A and B corresponded well to gene cluster A and B respectively, and showed significantly different prognoses and correlations with various clinical parameters (Figures 4B,C). Similarly, almost all IRGs showed differences in expressions between gene cluster A and B (Figure 4D). ICD scores were assigned to the samples according to the levels of expression of prognostic DEGs (Supplementary Table S13 **)**. Results of the survival curve analysis indicated that the low ICD score group showed significantly better survival relative to the high ICD score group (Figure 5A). The Sankey diagram presents the correspondence between ICD score, ICD clusters and ICD gene clusters (Figure 5B). The correlation between ICD cluster B and ICD gene cluster B with low ICD score showed a good prognosis as shown in Figures 5C,D; Supplementary Figure S1B. Additionally, as shown in Figure 5E, the ICD score was positively correlated with the infiltration of the vast majority of immune cells and negatively correlated with the infiltration of CD56 ^bright^ natural killer cells, CD56 ^dim^ natural killer cells, monocytes and type 17 T helper cells.

### 3.5 Relationship between the ICD score and TMB

**FIGURE 6:**
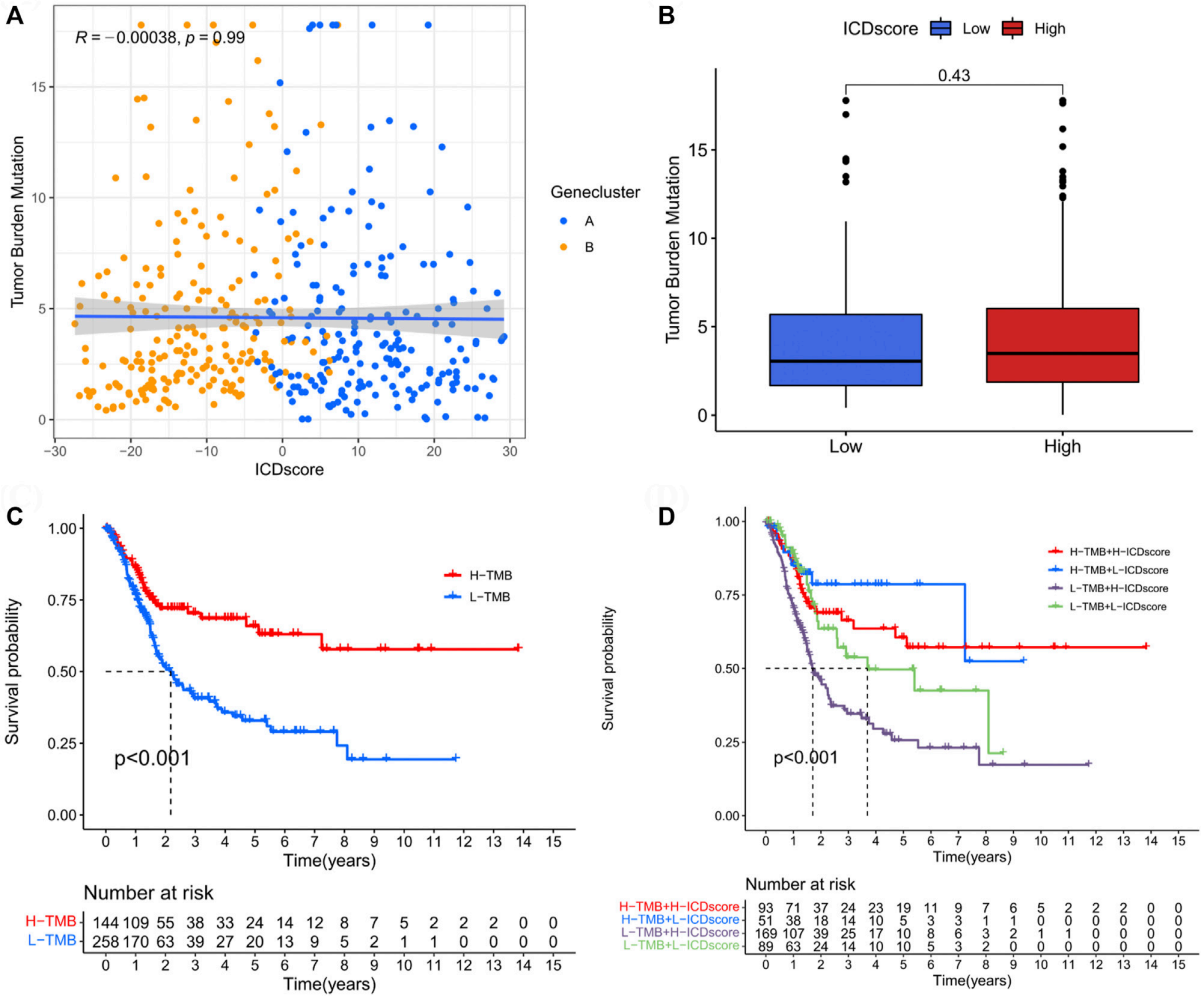
Relationship between ICD score and tumour mutation burden. **(A)** Correlation between ICD score and TMB in bladder cancer. **(B)** Differences in the TMB value between the different ICD score groups. **(C)** Kaplan–Meier curve analysis showing prognosis benefits of high TMB. **(D)** Kaplan–Meier curve analysis concerning the combination of ICD score and TMB.

No correlation was found between TMB and ICD score (Figures 6A,B), suggesting that the mechanism linking the ICD patterns to BCA did not affect TMB. Moreover, as there was a correlation between TMB and prognosis (Figure 6C **)**, the TMB group was integrated with the ICD score group and this combination was found to help predict survival probability; low TMB and high ICD score groups showed the worst prognosis, while high TMB and low ICD score group had the best prognosis (Figure 6D).

### 3.6 Correlation of ICD score with clinicopathological information

**FIGURE 7:**
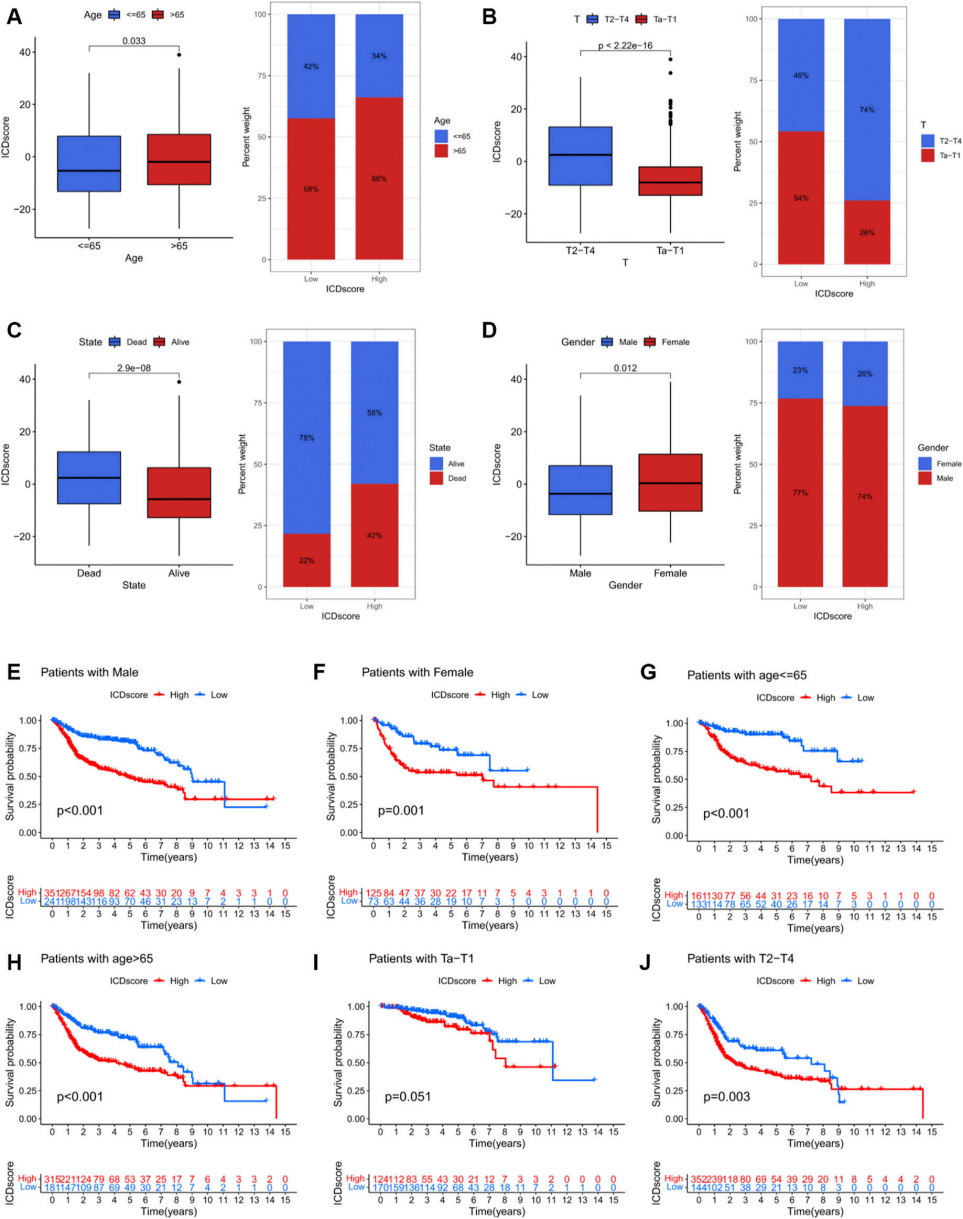
Relationship between ICD score and different clinical parameters and Kaplan–Meier survival analysis of different ICD scores in different subgroups of the merged cohort. **(A)** Relationships between ICD score and age. **(B)** Relationship between ICD score and tumour T stage. **(C)** Relationship between ICD score and alive/dead status. **(D)** Relationship between ICD score and gender. **(E)** Kaplan–Meier survival analysis in male patients. **(F)** Kaplan–Meier survival analysis in female patients. **(G)** Kaplan–Meier survival analysis in patients aged ≤ 65 years. **(H)** Kaplan–Meier survival analysis in patients aged > 65 years. **(I)** Kaplan–Meier survival analysis in patients with Ta–T1 stage disease. **(J)** Kaplan–Meier survival analysis in patients with T2–T4 stage disease.

We further examined the potential prognostic value of the ICD score by investigating its correlation with clinicopathological information (Supplementary Table S14). The results indicated that the ICD score was higher in patients in “Dead state”, “T2-T4”, “> 65 years” and “female” subgroups (Figures 7A–D). Furthermore, survival analysis confirmed that the ICD score could predict survival differences in multiple subgroups, including male, female, age ≤ 65 years, age > 65 years and T2-T4 subgroups (Figures 7E–J). These results demonstrated the prognostic predictive value of the ICD score for BCA patients with different clinicopathological conditions while elucidating the value of the ICD patterns for BCA.

### 3.7 Predictive value of the ICD score for immunotherapeutic responses

**FIGURE 8:**
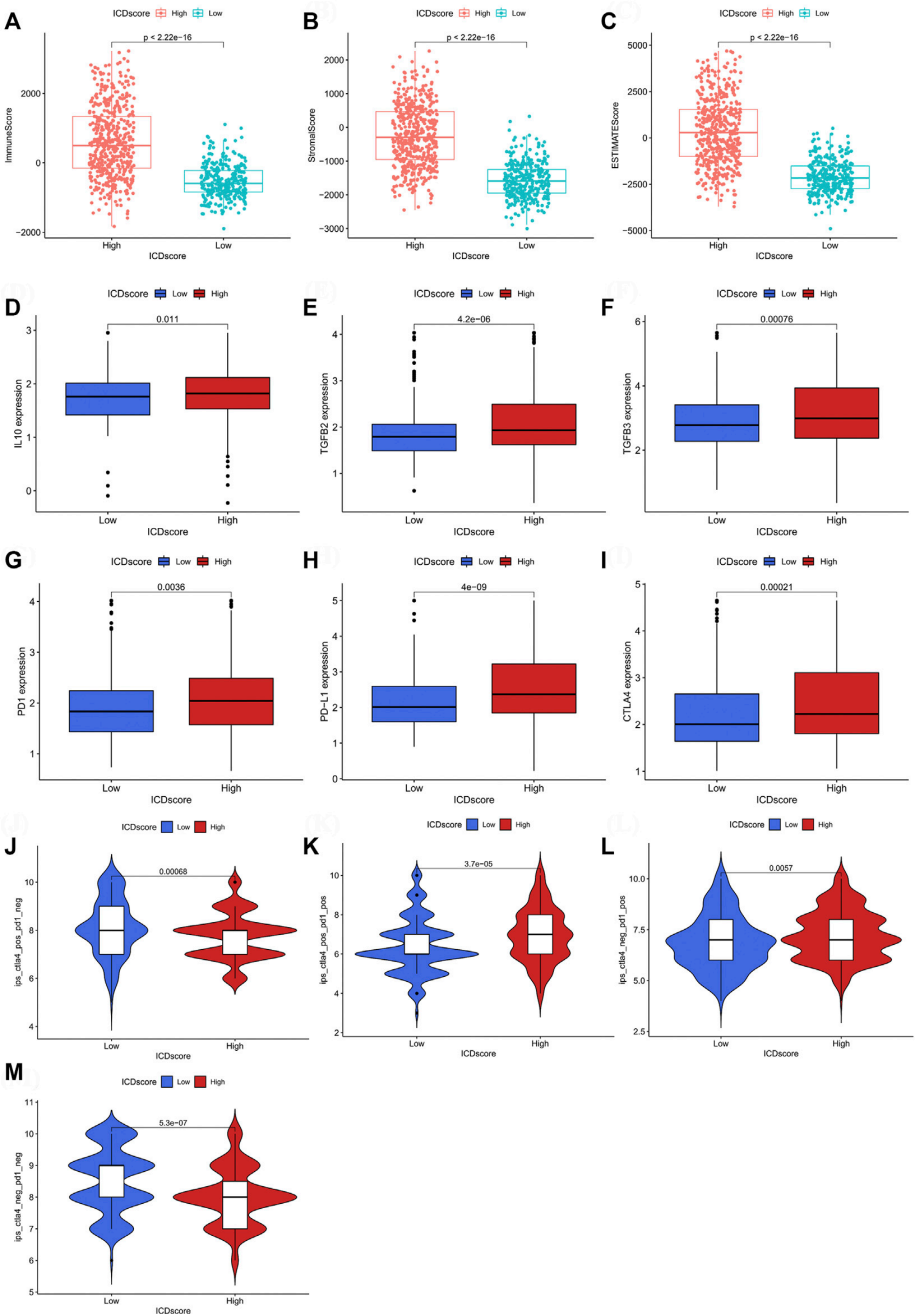
The indication of ICD score on immune microenvironment and prediction of immunotherapy response in bladder cancer. **(A–C)** ESTIMATE immune score between different ICD score groups. **(D–F)** Differences in the expression of IL10, TGFβ2 and TGFβ3 between different ICD score groups. **(G–I)** Differences in the expression of PD-1, PD-L1 and CTLA-4 between different ICD score groups. **(J–M)** Differences in the immunotherapeutic effects of four different strategies between the different ICD score groups.

We examined the relationship of the ICD score with the immune microenvironment using the R package “ESTIMATE”. In BCA, the immune, stromal and ESTIMATE scores were significantly different between patients with different clinicopathological features, and high stromal and ESTIMATE scores were associated with a poor prognosis **(** Supplementary Figure S3). The low ICD score group had lower immune, stromal and ESTIMATE scores relative to the high ICD score group (Figures 8A–C; Supplementary Table S15). These findings suggested that different ICD characteristics may affect the survival of patients by regulating the immune microenvironment. Further, we investigated whether the ICD score was a good indicator of the immunotherapeutic responses of patients with BCA. PD-1, PD-L1 and CTLA-4 expression levels in the high ICD score group were all significantly higher than those in the low ICD score group (Figures 8D–F), suggesting that the ICD score may be correlated with immunotherapeutic responses. To distinguish the therapeutic responses of different immunotherapy targets, the immunophenoscore (IPS) in the TCIA database (https://www.tcia.at/home; Supplementary Table S16) was analyzed. Interestingly, in CTLA-4_ pos + PD-1_ pos and PD-1_ pos + CTLA-4_ neg group, the high ICD score group showed a higher IPS than the low ICD score group, whereas in PD-1_ neg + CTLA-4_ neg and PD-1_ neg + CTLA-4_ pos group, the IPS results showed opposite trends (Figures 8J–M). These results suggested that patients in the low ICD score group may exhibit better responses to anti-CTLA-4 treatment, whereas those in the high ICD score group may show better responses to anti-PD-1/PD-L1 treatment or a combination treatment of anti-PD-1/PD-L1 and anti-CTLA-4. The above results suggested that the correlation between ICD and response to immunotherapy may be target specific. We also examined the differences in sensitivity towards traditional chemotherapeutic agents and molecular targets in different ICD score groups and ICD clusters. A total of 139 agents exhibited differential sensitivities (Supplementary Table S17), including tyrosine kinase inhibitors, epigenetic modifiers and traditional chemotherapeutic agents (Supplementary Figure S4).

### 3.8 Expression and functional analysis for CALR in BCA cell lines

**FIGURE 9:**
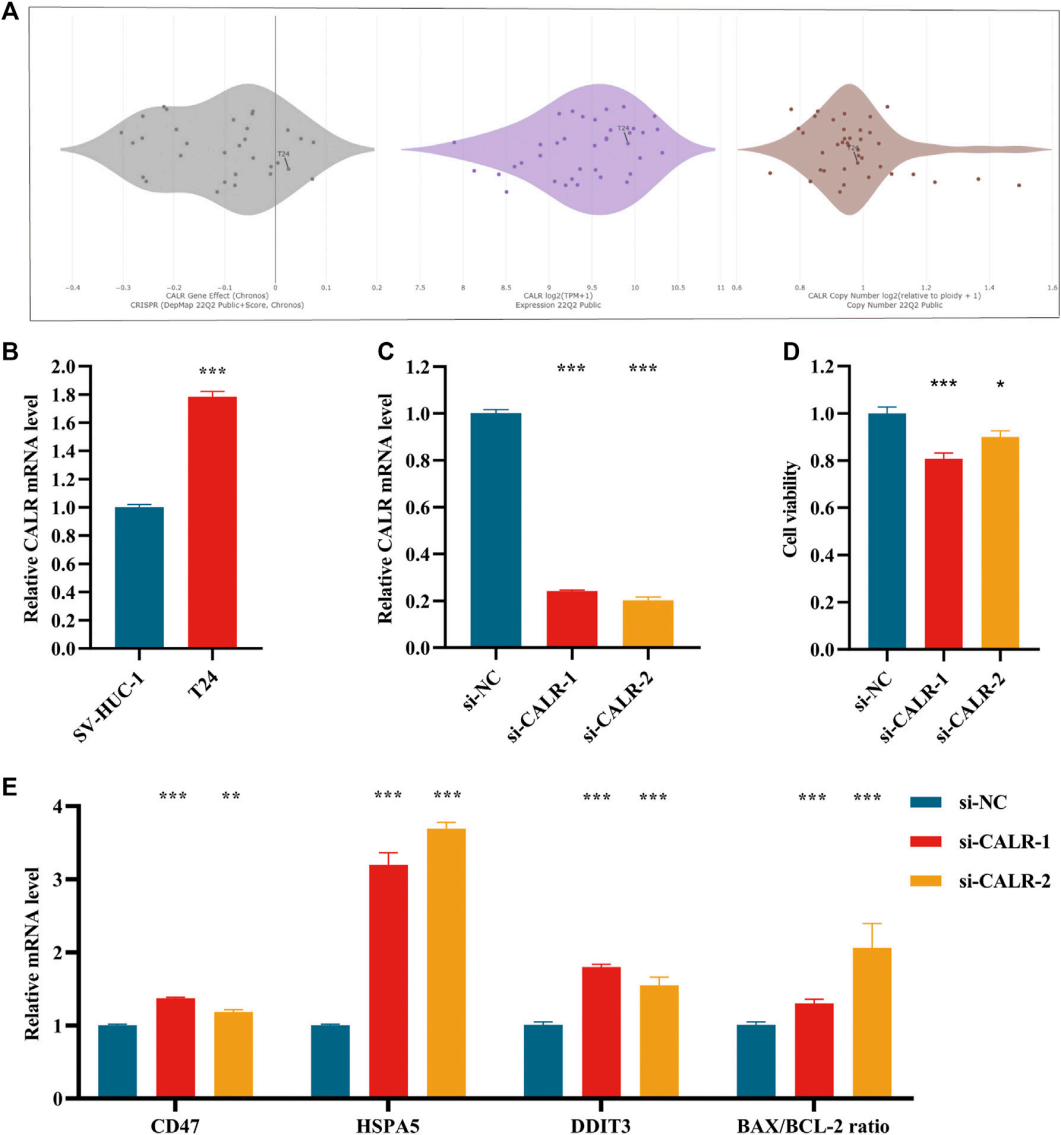
CALR expression in bladder cancer cell lines. **(A)** CALR expression of bladder cancer cell lines in different data sets in CCLE database. **(B)** Evaluation of the relative CALR/β-Actin mRNA expression levels, normalized with the SV-HUC-1 cell group. **(C)** Evaluation of the mRNA expression levels of CALR in si-NC, si-CALR-1 and si-CALR-2 T24 cells using quantitative PCR, with the expression levels normalized to those of *β*-ACTIN. **(D)** CCK8 assay was used for cell viability of si-NC, si-CALR-1 and si-CALR-2 T24 cells and normalized with the si-NC group. **(E)** Evaluation of the mRNA expression levels of CD47, HSPA5, DDIT3, BAX and BCL-2 in T24 cells treated with si-NC, si-CALR-1, or si-CALR-2. Experiments were performed in triplicates. All data are expressed as mean ± standard error (SE). The asterisk symbol indicated the statistical *p*-value. (*p < 0.05; **p < 0.01; ***p < 0.001).

The expression profile of CALR in BCA cells was queried on the CCLE database and the T24 cell line, highly expressed CALR in multiple datasets, was selected to examine the expression and function of CALR (Figure 9A). The normal human urothelial cell line, SV-HUC-1, was the control group. Results of the qRT-PCR analysis indicated that the T24 cells expressed higher mRNA levels of CALR than SV-HUC-1 cells (Figure 9B). CALR gene expression in si-CALR T24 cells was significantly lower than that in si-NC T24 cells, suggesting a successful CALR knockdown (Figure 9C). CCK8 assays showed that the viability of si-CALR T24 cells was significantly lower than that of si-NC cells (Figure 9D). Finally, the mechanisms of CALR knockdown that evoked inhibition of BCA cell viability were preliminarily investigated. Interestingly, the relative levels of CALR and CD47 determine the ultimate course of ICD; the mRNA expression of CD47 was upregulated to some extent after knocking down CALR. Additionally, CALR was involved in the proper functioning of the endoplasmic reticulum (ER). ER stress-related apoptotic pathways, BIP, CHOP and BAX/BCL-2 ratio were all significantly upregulated after knocking down CALR (Figure 9E).

## 4 Discussion

In the urinary tract, BCA is the second most common malignancy, second only to prostate cancer (Antoni et al., 2017). Although patients with early BCA have better outcomes with surgical treatment, recurrence is not uncommon and patients with advanced BCA often face tumor recurrence and metastasis even after treatment with a combination of chemotherapy and surgery (Berdik, 2017). Immunotherapy, as a new treatment modality for BCA, is currently considered a reliable second-line treatment; however, individual differences in efficacy limit its applications (Gómez de Liaño Lista et al., 2020). Previous studies showed that certain radiotherapeutic and chemotherapeutic measures were able to induce ICD in tumor cells, thus altering the TIME (Zhou et al., 2019). Moreover, chemotherapy-induced ICD has a synergistic effect with immunotherapeutic outcomes (Suresh et al., 2020). Therefore, it is of great value to reveal the correlation between ICD-related signatures and immunotherapy in BCA.

In the present study, IRGs were found to be closely related to the prognosis and TIME of BCA. Further, the BCA cohort was divided into two ICD clusters based on the expression profiles of IRGs. Patients in ICD cluster A showed higher ICD scores and more defined immune cell infiltrate profile and better responsiveness to immunotherapy; it was thus identified as an immune-hot tumor subtype despite the overall prognosis being slightly worse than ICD cluster B.

We combined four large BCA cohorts and examined the correlation between BCA and ICD-mediated patterns. The significant differences in the expression of IRGs between normal and tumor tissues and the higher CNV frequency for IRGs suggested their importance in BCA (Figures 1A,B). Twenty-eight IRGs were successfully identified in the combined cohort and 23 genes were found to be associated with the prognosis (Figures 2B–X). Further analysis of these IRGs also suggested that different ICD-mediated patterns did exist in BCA and these had better predictive significance for both prognosis and response to different immunotherapies and deserved further investigation. The two distinct ICD-mediated patterns had substantially different manifestations in BCA. The ICD cluster A patients had a slightly worse prognosis and the possible mechanisms were further elucidated by GSVA and ssGSEA. Results of the GSVA indicated that the ICD cluster A-enriched upregulated pathways mainly included three major classes (Supplementary Figure S3A). The first category was the pathways related to EMT and tumor migration ability, whereby their upregulation was closely related to the invasive capacity of tumors. The second category was the key signaling pathways involved in inflammation and cancer progression, represented by the nod-like receptor and the JAK-STAT signaling pathways, which could promote “uncontrollable inflammation” (Zhao et al., 2021) and contribute to a suppressive TIME. The third class was the chemokine signaling and leukocyte trans-endothelial migration pathways, which could coordinate immune cell trafficking to the tumor site, thereby remodeling the TIME and were generally responsible for the infiltration of tumor-promoting immune cells (Nagarsheth et al., 2017). That was, changes in invasive capacity and activation of uncontrollable inflammation with alterations in the TIME may contribute to the poor prognosis in patients of ICD cluster A. Two features of ICD cluster A-enriched GO terms (Supplementary Figure S3B) were: first, almost all of the terms were associated with immune cells; second, it was possible to both promote immunostimulation and induce immunosuppression. The contribution of enriched dendritic cells, neutrophils, monocytes, macrophages and other tumor-associated myeloid cells (TAMCs) to the TIME is uncertain (Dou and Fang, 2021). In particular, the two enriched pathways, negative regulation of lymphocyte migration and chemotaxis of lymphocyte migration acted in opposite directions. Given the aforementioned changes in immune cell function to ICD and the TIME, we performed GSEA for immune cell infiltration (Figure 3A). Except for monocytes, all 22 immune cell types showed significantly different abundances between the two ICD clusters and the vast majority of immune cells were enriched in ICD cluster A. High ICD scores corresponding to ICD cluster A were associated with higher immune and stromal scores (Figures 8A–C). We, therefore, reasoned that ICD cluster A was a class of “immune hot tumors”, wherein the TIME was more active, with abundant immune cell and TAMCs infiltration in the TIME. Despite that the tumor cells could attract a large number of CD8+T cells mediating ICD due to their immunogenic nature, the tumor cells could also recruit immune suppressive Treg cells to combat ICD. Thus, the complex TIME may contribute to the poorer prognosis of patients in the ICD cluster A.

As shown in Figure 2A, CALR, CASP8, TLR4 and LY96 were the four core genes most significantly associated with the prognosis of BCA patients (*p* < 0.01), with CALR and LY96 as risk factors while CASP8 and TLR4 as protective factors. Among these four IRGs, only CALR and TLR4 showed consistency between the expression and prognostic influence. For example, CALR was more highly-expressed in BCA patients than controls, meanwhile survival analysis showed CALR was a risk factor. So far, the role of TLR4 in BCA has been extensively explored (Lu et al., 2021), consistent with our results showing TLR4 was a protective factor. However, the correlation between BCA and CALR has been rarely reported. During ICD, CALR is exposed on the surface of the cell membrane and acts as an “eat me” signal that promotes the engulfment of dying tumor cells by dendritic cells or their precursors and CALR is normally localized in the endoplasmic reticulum (ER), although it would translocate to the surface of the cell membrane under stress (Gardai et al., 2005; Fucikova et al., 2020). Interestingly, the mRNA of CALR was not consistently expressed in different tumor cells. Increased CALR levels may imply a better or worse prognosis (Fucikova et al., 2021). We found increased CALR expression in BCA which correlated with poor prognosis (Figure 2B). This is consistent with all reported findings (Liu et al., 2020; Zhu et al., 2021). The results of our *in vitro* experiments validated that the mRNA expression of CALR was higher in BCA cells T24 than in normal urothelial cells SV-HUC-1 (Figure 9B); upon knocking down the gene expression of CALR in T24 cells, the viability of tumor cells reduced significantly (Figure 9D), a phenomenon consistent with the clinical outcomes. With regard to increased expression of CALR in BCA being associated with poor prognosis, we did the following to examine. First, the reason why the high expression of CALR results in poor prognosis of some tumors may stem from the compensatory overexpression of CD47, as this integrin-associated protein actively inhibits phagocytosis of dying cells. The relative levels of surface exposed CALR and CD47 together determine the ultimate course of ICD (Fucikova et al., 2021). However, the expression of CD47 increased slightly after knocking down CALR (Figure 9E), indicating that the above speculation did not apply to BCA. It has also been proposed that CALR acts as a key regulator of ER homeostasis and this may be partially required for tumor progression (Lu et al., 2015). Therefore, we detected the changes in ER stress-related pathways. Results of qRT-PCR analysis suggested that CALR knockdown caused a significant upregulation of ER stress markers (DDIT3 and HSPA5) and apoptotic markers (BAX/BCL-2). Similar findings have been reported in ovarian cancer previously (Kasikova et al., 2019). The above results suggested that elevated CALR levels in BCA may promote tumorigenesis by maintaining ER homeostasis in tumor cells. Increased CALR expression may suppress ER stress in tumor cells, thereby exerting an inhibitory effect on induced ICD.

To further examine the intrinsic features of different ICD clusters, two distinct gene clusters in the two ICD clusters were identified. These two gene clusters showed distinct survival outcomes and IRG expression profiles (Figures 4B,D). A clear difference was observed in the survival status, T stage, gender and ICD cluster distribution between the gene clusters (Figure 4C). Thus, differences between these gene clusters may help further screen for genetic differences in different ICD-mediated patterns of BCA. Correspondingly, we developed an ICD score to quantify the ICD-mediated pattern in individual samples. The results confirmed that patients in the high ICD score group had a worse prognosis (Figure 5A). Further analysis showed that a high ICD score was a common feature of both ICD cluster A and gene cluster A; there was a consistency in the poor prognostic outcomes of the three (Figures 5B–D). Lower ICD scores were obtained in BCA patients of the following subgroups: alive with disease, Ta-T1, age ≤ 65 and male (Figures 7A–D). The ICD score was valuable in predicting survival outcomes in the following groups: age ≤ 65 years (*p* < 0.001), age > 65 years (*p* < 0.001), female (*p* = 0.001), male (*p* < 0.001) group, Ta-T1 (*p* = 0.051) and T2-T4 (*p* = 0.003) (Figures 5E–J). The above results further validated the value of the ICD score for prognostic prediction. Additionally, although we did not find a correlation between the ICD score and TMB, ICD score combined with the TMB grouping helped make a better prognostic judgment (Figure 6).

Most importantly, we analyzed the correlation of the ICD score with TIME and immunotherapeutic responses. First, we analyzed the correlation of the ICD score with the ESTIMATE score and found that the high ICD score group showed higher levels of stromal and immune cell infiltration (Figures 8A–C), corroborating that the high ICD score group corresponds to the “immune hot tumors”. The detailed characteristics of TIME for different ICD score groups were further analyzed. Currently, research holds that immunosuppressive cytokines such as IL-10 can directly act on natural killer (NK) cells and inhibit their function and proliferation (Mirlekar, 2022). The high ICD score group correlated positively with the infiltration of most immune cells, while negatively with NK cells and monocytes responsible for tumor killing (Figure 5E). We simultaneously found that cytokines involved in the immunosuppressive processes (IL-10, TGF-β2 and TGF-β3) were also elevated in the high ICD score group (Figures 8D–F). Immune checkpoint genes (PD-1, PD-L1 and CTLA-4) were also elevated in the high ICD score group (Figures 8G–I). The above results indicated that the high ICD score group had the property of inhibiting or evading immune responses. And alterations in this type of TIME were able to influence the efficacy of multiple immunotherapies including chimeric antigen receptor T cell therapy (Titov et al., 2022).

Previous studies confirm that enrichment of CD4^+^ and CD8^+^ T cells and high expression of immune checkpoint genes in tumor tissues are generally associated with a good response to anti-PD-1/PD-L1 therapy (Chen and Mellman, 2017; Morad et al., 2021). The IPS prediction results were consistent with this view: patients in the high ICD score group may show a better response to anti-PD-1/PD-L1 treatment or the combination of anti-PD-1/PD-L1 and anti-CTLA-4 (Figures 8J,K). This is in close agreement with the characteristic high expression of immune checkpoint genes and “immune hot tumors” in the high ICD score group. Interestingly, patients in the low ICD score group may show a better response to anti-CTLA-4 treatment (Figure 8L). Similarly, it was confirmed that baseline tumor immune cell infiltration status could not predict responses to anti-CTLA-4 therapy for the following reasons: unlike anti-PD-1/PD-L1 therapy, which mainly acted on the tumor cells, anti-CTLA-4 therapy acts on T cells of the body, and the immune microenvironment in the tumor region might not be the best predictor of responses to anti-CTLA-4 therapy (Huang et al., 2011). Taken together, the ICD score was a good indicator for predicting the efficacy of ICIs. As a new cancer adjuvant therapy, the induced ICD therapy is not a stand-alone option; radiotherapy, chemotherapy, epigenetic modifiers, targeted therapies and oncolytic viral therapy, all have the potential to induce ICD to exert anticancer effects, especially partial tyrosine kinase inhibitors, epigenetic modifiers and traditional chemotherapy drugs (Fucikova et al., 2020). We, therefore, examined differences in sensitivity between traditional chemotherapeutic agents and molecular targets across different ICD score groups and ICD clusters. A total of 139 drugs showed varying sensitivity among different ICD score groups and ICD clusters. The ICD cluster A and high ICD score groups showed greater infiltration of CD8^+^ T cells (Figure 3A, Figure 5A), thus, we hypothesized that there was a greater potential for benefiting from induced ICD therapies. The results were also indeed consistent with our notion that patients in the high ICD score group and ICD cluster A group were more sensitive to most of these drugs (Supplementary Figure S4).

Our study has the following limitations: we conducted an initial examination using TCGA and three GEO cohorts and further inclusion of more multicenter cohorts for external validation was warranted. We only performed *in vitro* experiments and further complementing these results with *in vivo* experiments could help elucidate the pathogenic mechanisms of key ICD genes in BCA. Additionally, the reasons why ICD scores differed in predicting the efficacy of anti-CTLA-4 versus anti-PD-1/PD-L1 also deserved further investigation.

## 5 Conclusion

In summary, we identified the existence of distinct ICD-mediated patterns in BCA, which were closely associated with the remodeling of the TIME. By concordance clustering analysis, ICD cluster A, with a high ICD score, belonged to the “immune hot tumor” subtype and these patients were more likely to benefit from ICIs and therapies that induce ICD. Further in-depth examination of ICD-related signatures was warranted to provide a broader outlook for therapeutic innovation and improved prognosis for BCA.

## Data Availability

The original contributions presented in the study are included in the article/Supplementary Material, further inquiries can be directed to the corresponding author.
