# Supplementary figures and images for: Immunogenic cell death mediation patterns reveal novel paradigm for characterizing the immune microenvironment and immunotherapeutic responses in bladder cancer
Source: Front Genet. 2022 Oct 25;13:1035484. doi: 10.3389/fgene.2022.1035484 (PMC9640952; doi:10.3389/fgene.2022.1035484)

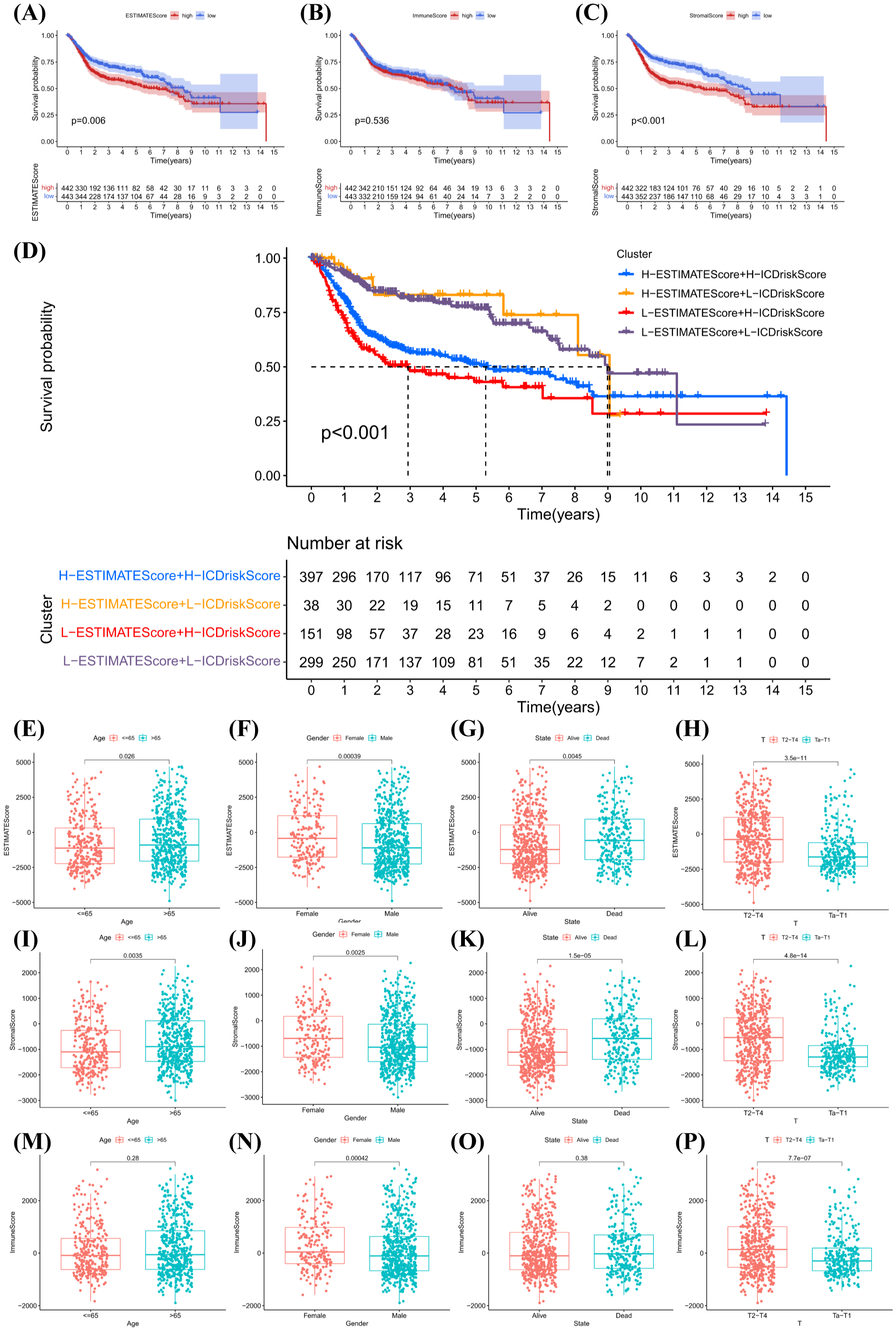

Supplement: Supplementary file 1 [file Image3.TIF]

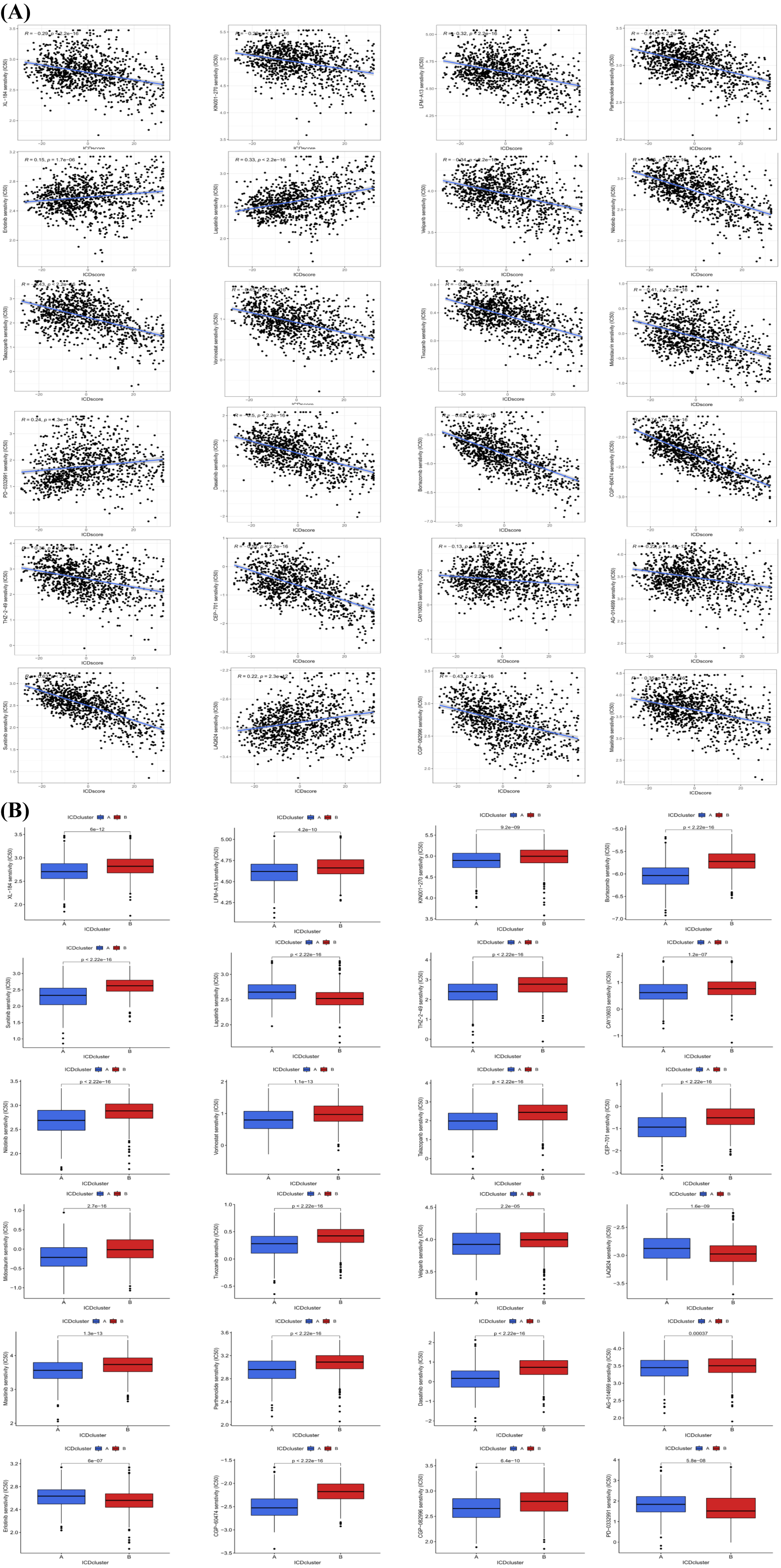

Supplement: Supplementary file 2 [file Image4.TIF]

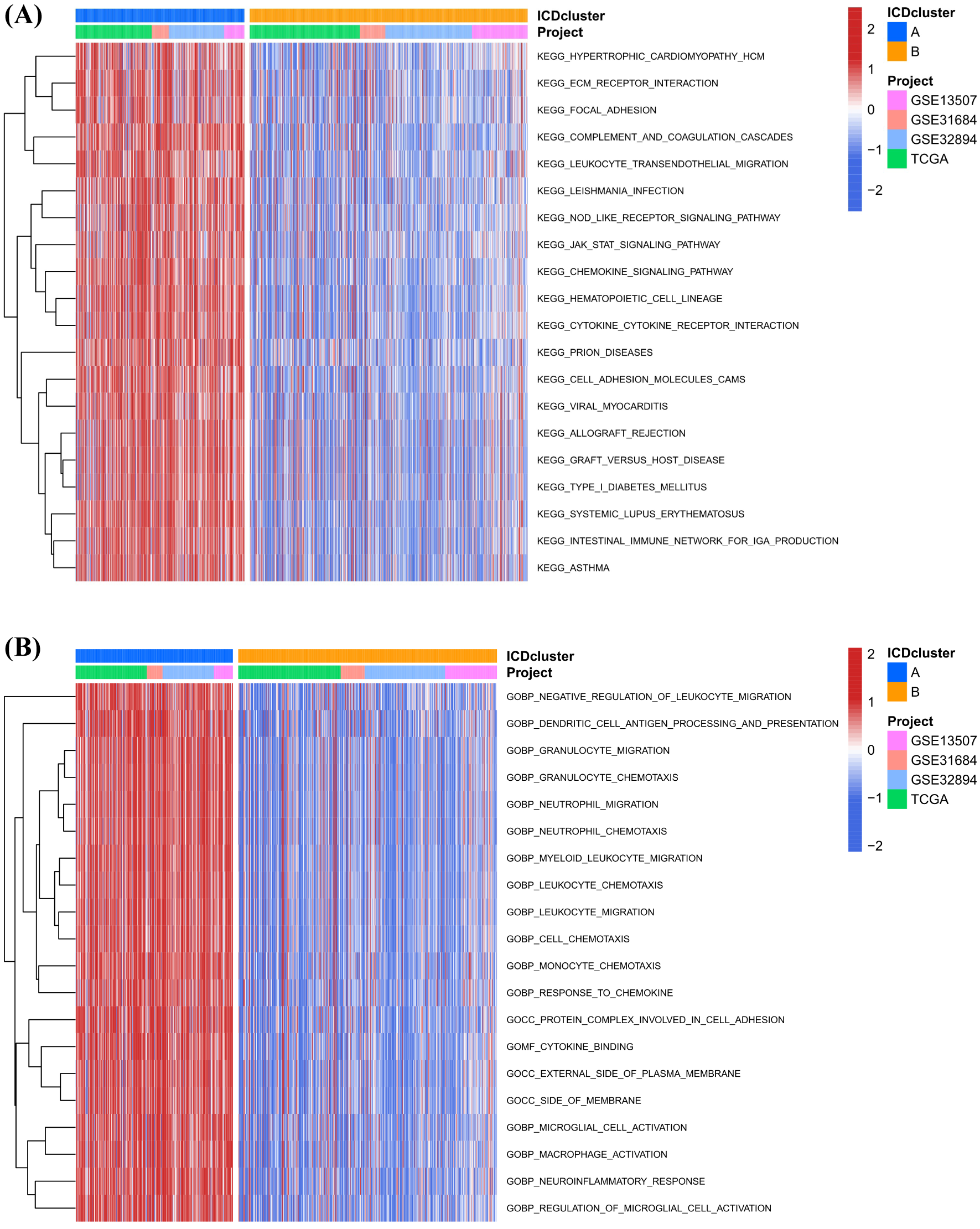

Supplement: Supplementary file 4 [file Image2.TIF]

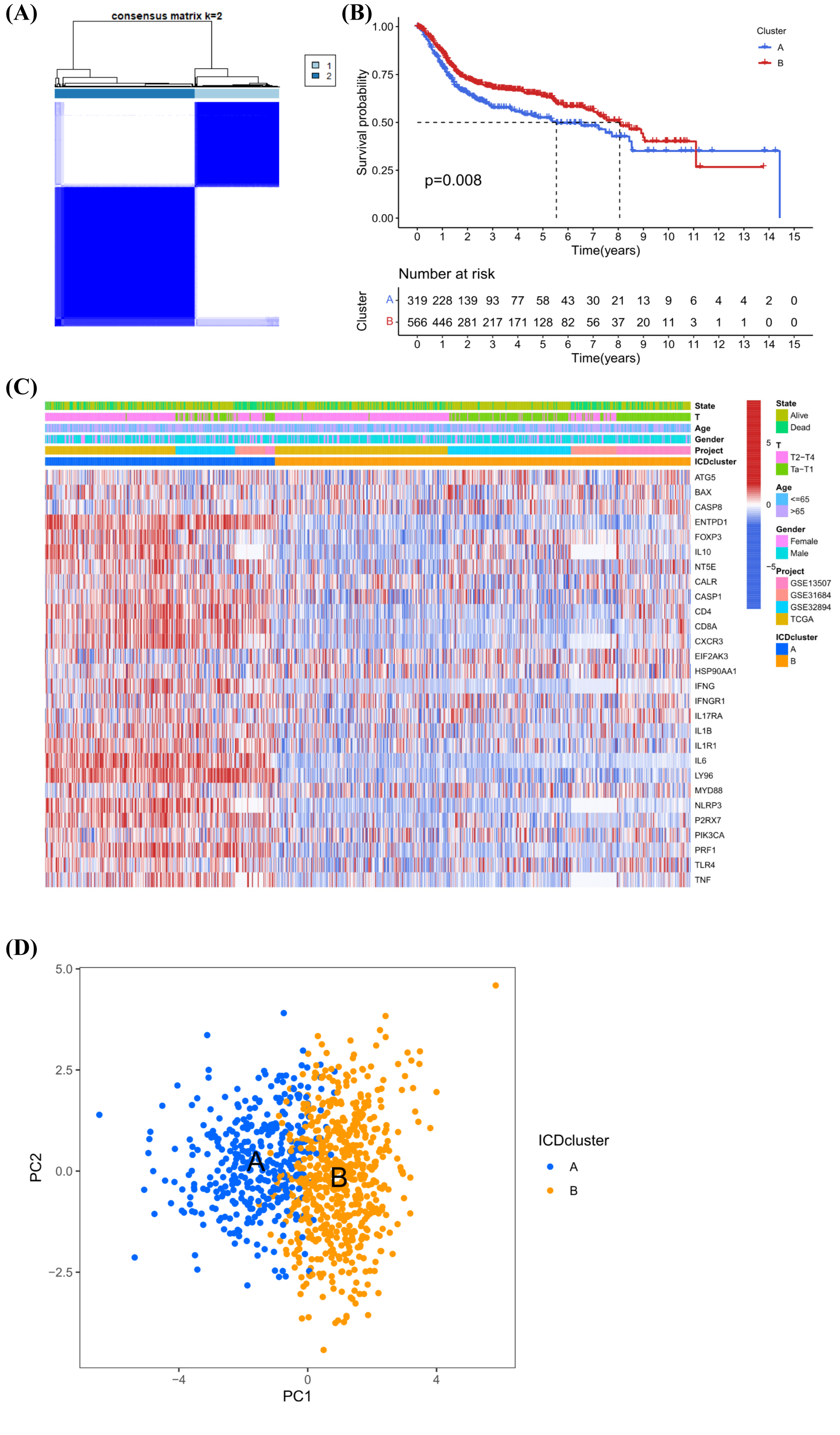

Supplement: Supplementary file 5 [file Image1.TIF]
